# Reactome and ORCID—fine-grained credit attribution for community curation

**DOI:** 10.1093/database/baz123

**Published:** 2019-12-04

**Authors:** Guilherme Viteri, Lisa Matthews, Thawfeek Varusai, Marc Gillespie, Marija Milacic, Justin Cook, Joel Weiser, Solomon Shorser, Konstantinos Sidiropoulos, Antonio Fabregat, Robin Haw, Guanming Wu, Lincoln Stein, Peter D’Eustachio, Henning Hermjakob

**Affiliations:** 1 European Molecular Biology Laboratory, European Bioinformatics Institute (EMBL-EBI), Wellcome Trust Genome Campus, Hinxton, Cambridgeshire CB10 1SD, UK; 2 Department of Biochemistry, NYU School of Medicine, New York, NY 10016, USA; 3 Ontario Institute for Cancer Research, Toronto, ON, M5G 0A3, Canada; 4 College of Pharmacy and Health Sciences, St. John’s University, Queens, NY 11439, USA; 5 Oregon Health and Science University, Portland, OR 97239, USA; 6 State Key Laboratory of Proteomics, Beijing Proteome Research Center, Beijing Institute of Lifeomics, National Center for Protein Sciences (The PHOENIX Center, Beijing), 102206, Beijing, China

## Abstract

Reactome is a manually curated, open-source, open-data knowledge base of biomolecular pathways. Reactome has always provided clear credit attribution for authors, curators and reviewers through fine-grained annotation of all three roles at the reaction and pathway level. These data are visible in the web interface and provided through the various data download formats. To enhance visibility and credit attribution for the work of authors, curators and reviewers, and to provide additional opportunities for Reactome community engagement, we have implemented key changes to Reactome: contributor names are now fully searchable in the web interface, and contributors can ‘claim’ their contributions to their ORCID profile with a few clicks. In addition, we are reaching out to domain experts to request their help in reviewing and editing Reactome pathways through a new ‘Contribution’ section, highlighting pathways which are awaiting community review.

Database URL: https://reactome.org

## Introduction

Reactome is a manually curated, open-source, open-data knowledge base of biomolecular pathways (1,2). The central element of Reactome is a biochemical reaction, with multiple reaction types, for example classical enzymatic reactions, translocations, complex formation and protein modifications. Reactions are linked by shared molecular entities into pathways, which in turn are grouped into a pathway hierarchy that is co-ordinated with the ‘Biological Process’ branch of the Gene Ontology (3).

The Reactome curation process is similar to the writing of a pathway review for a scientific journal. Typically, we recruit an external domain expert for the target pathway, the pathway author. This expert collaborates with the professional Reactome curator in creating a representation of the pathway in the Reactome database, potentially in several iterations. While this work often starts from reviews, as a key requirement, we trace back and link assertions to the underlying primary literature. In cases where published information needed to annotate a reaction fully is not available, the resulting event is clearly marked as a ‘Black box’ reaction. The resulting pathway representation is then reviewed internally by a senior curator. Next, we recruit a second external domain expert, from a different group, as a reviewer, who provides major or minor comments and corrections, triggering an update of the pathway representation. Once all three key people (author, curator, and reviewer (contributors)) are satisfied, the pathway is released in the next Reactome quarterly release. While this process is slow and labour-intensive, it assures a high quality of Reactome content.

A Reactome curator can assume the role of the expert author or reviewer, typically when they have worked in the target domain previously. However, the recruitment of authors and reviewers is currently the major bottleneck in the Reactome curation process: on average, we are contacting 10 domain experts for one actual contributor. This is not really surprising; domain experts are providing their expertise and time voluntarily, without payment. Sometimes, we achieve a scientific publication with the authors/reviewers as co-authors (4), but often this is not possible. Then, the only recognition of their work is credit attribution through the Reactome records they have authored or reviewed. Even for professional (paid) curators, their scientific contribution is mainly visible through Reactome records, their productivity in terms of scientific publications is typically only visible in the Reactome consortium database publications like (1,5). Recognising this challenge, recently there are significant efforts to improve citability and scientific credit attribution for non-publication objects like datasets (6,7).

Reactome has always provided clear credit attribution for authors, curators and reviewers through fine-grained annotation of all three roles at the reaction and pathway levels. These data are visible in the web interface and provided through the various data download formats. Starting in 2008 and retroactively implemented for earlier releases, on author/reviewer request, we also provide DOIs (8) for pathways to make them citable in scientific publications or on contributor’s CVs and similar documents. However, DOIs are only created at the pathway level. In addition, Reactome is a dynamic knowledge base, and changing science or content reorganisations require relatively frequent changes of existing pathways, which is not fully compatible with the DOI concept of immutability for objects referenced by a DOI.

## Results

**Figure 1:**
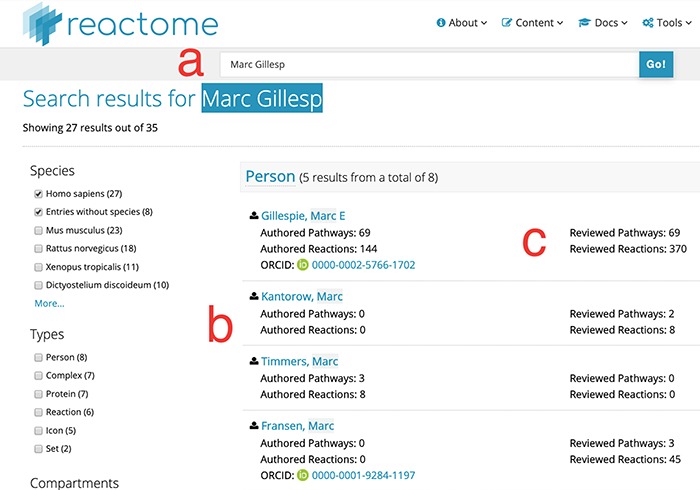
Search results for partial author name ‘Marc Gillesp’. Direct access URL https://reactome.org/content/query?q=Marc+Gillesp&species=Homo+sapiens&species=Entries+without+species&cluster=true, accessed 2019/08/15. Within the figure, label ‘a’ marks the mis-spelt contributor name, label ‘b’ marks the list of other contributor names matching the search terms and label ‘c’ marks the fine-grained ‘contribution matrix’, distinguishing between pathways and reactions in one dimension and authoring/reviewing in the other dimension.

**Figure 2:**
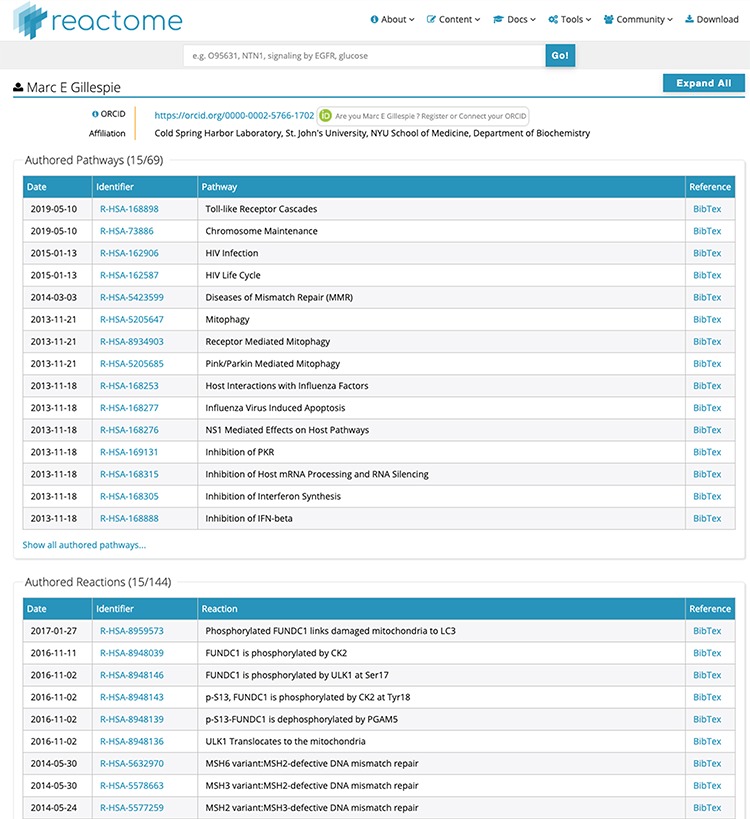
Detailed list of contributions for ‘Marc E Gillespie’. Unique identifiers in the ‘Identifier’ column directly link to the relevant pathway/reaction. BibTex records can be downloaded for each data object if desired. After ORCID authentication at the top of the page, all contributions can be claimed to the contributor’s ORCID record with a single click, or individual data object can be claimed for finer granularity.

**Figure 3:**
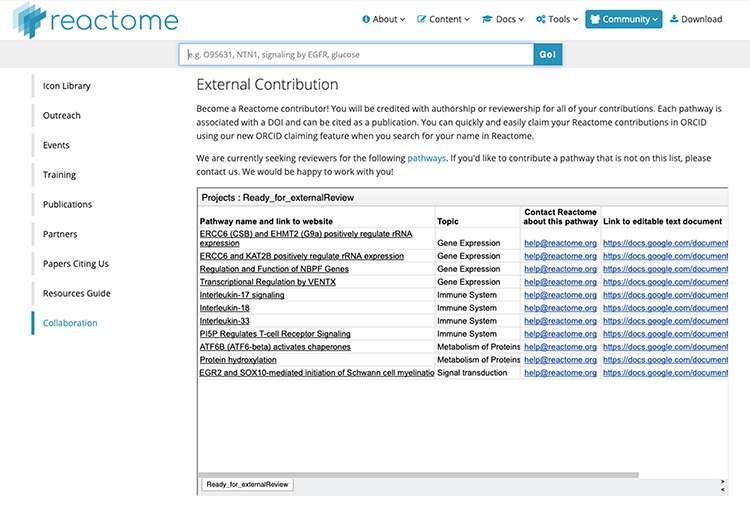
Reactome ‘External Contribution’ page. Direct access URL https://reactome.org/community/collaboration, accessed 2019/08/15. This page lists pathways which have passed internal validation, and for which we are eager to find an external reviewer. The links in the leftmost column show the preliminary pathway in the Reactome ‘reviewer view’, which is slightly simpler than the final web view. The links in the rightmost column lead to text documents which can be copied for local editing. We would like to encourage any domain experts to contribute to the correct representation of ‘their’ pathways by contributing to these pending reviews.

To enhance visibility and credit attribution for the work of authors, curators and reviewers, and to provide additional opportunities for Reactome community engagement, we have implemented three key changes to the Reactome web interface:

(i) Searchability: contributor names are now fully searchable in the standard Reactome web interface, with the same advanced features as other data objects, including auto-completion and approximate search. As illustrated in Figure 1, label a, the search for a unique name returns a result, even if the name is incomplete, here ‘Marc Gillesp’ instead of ‘Marc Gillespie’. In case of multiple matching contributors, all are listed and presented as part of the faceted search results as a separate facet, similar to other data objects (Figure 1, label b). This search functionality is distinct from the search for authors of publications cited by Reactome. If a user searches for the name of an author of a publication cited in Reactome, the web interface will return the data objects, for example pathways, which cite the publication(s) of the author. In contrast, contributor names are explicitly listed as separate data objects. For each contributor, their contribution is listed in a fine-grained matrix, distinguishing between pathways and reactions as well as between author and reviewer roles (Figure 1, label c). Clicking on the contributor name returns a new page with a detailed view of the work attributed to the Reactome contributor (Figure 2). Importantly, if the contributor has provided their ORCID identifier, the identifier is clearly shown as part of the record and linked to their ORCID profile. ORCID provides unique identifiers for scientists, allowing to disambiguate names and aggregate scientific contributions like publications and datasets to a central ORCID profile.

(ii) ORCID claiming: where the contributor has provided an ORCID identifier, the top of the detailed contribution page (Figure 2) shows a link that allows the contributor to validate themselves using the ORCID API. After validation, the details page provides additional buttons to claim, at a single click, all their Reactome contributions to their ORCID profile, or provide a more fine-grained selection of the Reactome contributions they would like to claim to their ORCID profile. In coordination with ORCID, the type of contribution for Reactome content is ‘data-set’. As of August 2019, 1473 Reactome pathways (64% of 2287 pathways in Reactome) and 6217 reactions (49% of 12 608 reactions in Reactome) have been claimed in ORCID. The lower claim rate for reactions is probably due to the fact that some large-scale contributors only claim pathways, to avoid filling their ORCID profile with many claims for individual reactions.

(iii) Community outreach for reviewer recruitment: By now, Reactome has reached a fairly good coverage of human pathway space. Although Reactome curation is as open-ended as research on human biology, we now typically add or update smaller pathway subsections, rather than entire new pathways. This exacerbates the problem of recruiting reviewers, as we need relatively more reviewers to review relatively smaller entities. In an experiment to increase Reactome user engagement and contribution, we have added a specific ‘Collaboration’ section to the Reactome ‘Community’ web pages (Figure 3). Here, we list pathways which have passed the internal review, but still require an external review to be ready for release. We expect that this opportunity might encourage Reactome users to become contributors and hopefully will also develop into a forum for larger-scale community contributions and both suggestions of future pathway curation projects and offers of pathway authorship.

## Discussion

Community contribution is a major or minor component of most biomolecular database resources. Sometimes, it is seen as an approach to replace expensive manual curation, though this is unlikely to be practical. Even resources with a strong emphasis on community curation like WikiPathways (9) or Pfam (10) strongly rely on professional curators to ensure database consistency and to provide major parts of the contents. However, in our experience, community contribution is essential to ensuring the quality and coverage of a complex knowledge base like Reactome. Unfortunately, the voluntary contribution of domain experts, as well as the work of curators, is often not realised or appreciated by the community. The strong community contribution to Reactome is, in our experience, not very well known, and yet Reactome critically depends on such contributions. Since 2002, 817 individuals have contributed to Reactome, but less than 30 of these are current or former paid curators, all the others are voluntary contributors whom we would like to strongly credit for their work. Reactome has always provided the names of content contributors at both reaction and pathway levels and since 2008 has provided DOIs for pathways.

ORCID offers scientists the possibility to claim a broad range of scientific outputs to their ORCID profile, and several data resources, for example Pfam and OmicsDI, now offer their users the possibility to claim annotations and datasets, respectively, to their ORCID profiles in a user-friendly manner (10,11). Here, we have presented a set of improvements to the Reactome web interface, including use of the ORCID API, to facilitate searching for an individual’s contribution to Reactome content, as well as claiming such content to an individual’s public ORCID profile, in an easy and fine-grained manner. With these measures, we aim to facilitate and improve credit attribution for Reactome content contributions. We also encourage more community contribution to Reactome content through a new section providing concrete requests from Reactome for external review.

In the context of the development of community standards for data citation (6), as well as nascent credit attribution and impact metrics for non-manuscript scientific output like datasets (12), we hope to contribute a small step towards a more multidimensional view of scientific productivity, where a scientist is more than their h-index.

## Author contributions

G.V., K.S. and A.F. developed the improvements to the Reactome web interface. T.V. and L.M. developed the Community Collaboration section. R.H., H.H., P.D. and L.S. conceived the project. P.D. coordinated the Reactome curation. R.H. and M.G. managed the Reactome DOI integration. H.H. coordinated this project and wrote the manuscript. All authors read and approved the manuscript.
